# Nutrition‐sensitive agriculture programme impacts on time use and associations with nutrition outcomes

**DOI:** 10.1111/mcn.13104

**Published:** 2020-12-10

**Authors:** Mara van den Bold, Lilia Bliznashka, Gayathri Ramani, Deanna Olney, Agnes Quisumbing, Abdoulaye Pedehombga, Marcellin Ouedraogo

**Affiliations:** ^1^ Poverty, Health and Nutrition Division International Food Policy Research Institute (IFPRI) Washington D.C. USA; ^2^ Department of Global Health and Population, Harvard T.H. Chan School of Public Health Harvard University Boston Massachusetts USA; ^3^ SELEVER Project AFRICSanté Bobo‐Dioulasso Burkina Faso; ^4^ Projet d'amélioration de l'Alimentation de la nutrition et de l'Hygiène en milieu familial (PAH/GIZ) Deutsche Gesellschaft für Internationale Zusammenarbeit Ouagadougou Burkina Faso

**Keywords:** agriculture, Burkina Faso, children, nutrition, time use, women's health

## Abstract

Success of nutrition‐sensitive agriculture programmes targeted to women may be influenced by increased demands on women's and other household members' time and by time‐related trade‐offs to accommodate programme participation. However, evidence of how such programmes impact time use and whether changes in time‐related demands negatively influence maternal or child health and nutrition outcomes is limited. This paper examines the impact of Helen Keller International's Enhanced Homestead Food Production programme in Burkina Faso (2010–2012) on women's and men's time use and associations between changes in women's time use and maternal and child health and nutrition outcomes. We used quantitative data from a cluster‐randomized controlled trial (baseline 2010, endline 2012) and qualitative data from two rounds of process evaluation (2011, 2012). Two‐stage analyses were used to first assess programme impacts on women's and men's time use using difference‐in‐difference impact estimates and second to evaluate whether programme impacts on women's time use were associated with changes in women's and children's health and nutrition outcomes. Programme impacts were considered significant if corrected *P* < 0.01, and associations were considered significant if *p* < 0.05 and *p <* 0.01. Qualitative data were analysed through manual coding and by calculating the means and standard deviations for the time spent by women and men on activities in intervention and control groups. Findings show that the programme significantly increased the amount of time women spent on agriculture in the intervention compared to the control group, but this was not associated with changes in maternal or child health or nutrition outcomes. Process evaluation data supported these findings.

**Key messages**

- Evidence of how nutrition‐sensitive agriculture programs targeted to women impact time use and how changes in time use affect nutrition outcomes is limited.
- An evaluation of a nutrition‐sensitive agriculture program in Burkina Faso significantly increased the time women spent on agriculture and improved maternal and child nutrition outcomes.
- Despite increasing the time women spent on agriculture, there was no evidence that this contributed to deleterious effects on their own or their children's nutrition.
- Future research should also look at these relationships and further include considerations of the impacts of nutrition‐sensitive agriculture programs on time use patterns of other household members.

## 1 INTRODUCTION

There is a growing interest in understanding how nutrition‐sensitive agriculture programmes in lower‐ and middle‐income countries (LMICs) affect maternal and child health and nutrition outcomes. These programmes are posited to reduce maternal and child undernutrition through multiple pathways, such as food intake, income, price dynamics and women's participation in agriculture (Ruel & Alderman, 2013; Ruel, Quisumbing, & Balagamwala, 2018). Nutrition‐sensitive agriculture programmes, such as those dedicated to homestead food production, often target women given their central role in their own and their children's health and nutrition. However, an important consideration in targeting these programmes to women is how the programme may affect demands on their time as well as that of other household members and how these changes in time use may affect their own or their children's health and nutrition outcomes.

Evidence from impact evaluations in LMICs suggests that nutrition‐sensitive agriculture programmes can improve both maternal and child nutrition outcomes (Berti, Krasevec, & FitzGerald, 2004; Girard, Self, McAuliffe, & Olude, 2012; Masset, Haddad, Cornelius, & Isaza‐Castro, 2012; Olney et al., 2016; Olney, Pedehombga, Ruel, & Dillon, 2015; Osei et al., 2015; Ruel et al., 2018). These studies suggest that impacts on intermediary outcomes such as improvements in production of nutrient‐rich foods, maternal knowledge and empowerment and infant and young child feeding (IYCF) practices likely contribute to programme health and nutrition impacts (Heckert, Olney, & Ruel, 2019; Johnston, Kadiyala, Stevano, Malapit, & Hull, 2015; Olney et al., 2015; Osei et al., 2015; Ruel & Alderman, 2013; van den Bold, Quisumbing, & Gillespie, 2013; van den Bold et al., 2015). In addition, when nutrition and health behaviour change communication (BCC) interventions are included, nutrition‐sensitive agriculture programmes tend to be more effective in achieving positive nutrition outcomes for women and children, and they tend to have greater impacts on child nutritional status when they include health and water, sanitation and hygiene interventions and micronutrient‐fortified products (Ruel et al., 2018). However, thus far, there is limited evidence on how these types of programmes impact women's and men's time use and how programme impacts on women's time use are associated with child and maternal health and nutrition outcomes.

Time use data are important for analysing how individuals within a household allocate their time to different activities. It is also important for assessing how time poverty—the burden of competing demands on an individual's time (Hirway, 2010)—may require them to make trade‐offs between so‐called productive (paid) and reproductive (e.g., domestic tasks and childcare) activities that may affect their own or their children's well‐being in different ways (Blackden & Wodon, 2006; Johnston et al., 2015; Stevano et al., 2018). Recent work examining time use as an agriculture‐nutrition pathway in LMICs finds that women play an important role in agriculture, as reflected in their commitments to agricultural activities, whether as farm workers or as farmers (Johnston, Stevano, Malapit, Hull, & Kadiyala, 2018; Stevano et al., 2018), and that women are important actors in the uptake of and response to agricultural interventions (Johnston et al., 2018). The way in which nutrition‐sensitive agriculture interventions impact time spent on agricultural activities is highly diverse, possibly due to the different ways in which changes in time burdens are managed, such as by extending the work day, reducing time spent on other activities and delegating tasks to other household members, or factors such as seasonality or household socio‐economic status that might influence time spent on different activities (Johnston et al., 2018; Stevano et al., 2018). Additionally, time use as a mediating factor in nutrition‐sensitive agriculture interventions may vary depending on socio‐economic status because wealthier households may have better access to assets that reduce workload.

Existing research on the gendered nature of time use in LMICs shows that men and women often allocate time differently. Furthermore, it shows that the gendered ways in which time is apportioned can be influenced by factors such as marital status, seasonality, access to social services, technology, infrastructure and, particularly, socio‐cultural norms that underpin gendered divisions of labour, which may interact in different ways depending on the historical, political, cultural and economic context (Johnston et al., 2015; Komatsu, Malapit, & Theis, 2015). To date, data have indicated that, generally, women work more total hours than men when time spent on reproductive activities is taken into consideration (Apps, 2003; Blackden & Wodon, 2006; FAO, 2009; Ilahi & Bank, 2000; World Bank, 2001). In addition, whereas men often perform tasks sequentially, women often perform productive and reproductive work at the same time, facing stricter trade‐offs between these activities or between different kinds of productive activities (Blackden & Wodon, 2006; Johnston et al., 2015). Women are generally also disproportionately responsible for reproductive tasks, such as food preparation and childcare (Johnston et al., 2015 , 2018). In rural sub‐Saharan Africa in particular, workloads are high and the differences in time allocation by gender are especially pronounced, as women are often responsible for tasks around the home such as cooking, child care and collecting water and firewood, and men are relatively more engaged in self‐employment or wage work (Blackden & Wodon, 2006; Ilahi & Bank, 2000). Based on the evidence that women in agricultural settings tend to face stricter trade‐offs in their time use, there is a risk that nutrition‐sensitive agriculture programmes targeted to women may exacerbate women's time conflicts and may negatively affect their own and their children's health and general well‐being.

In this paper, we assess the impact of Helen Keller International's (HKI) Enhanced Homestead Food Production (EHFP) programme implemented from 2010 to 2012 in eastern Burkina Faso on women's and men's time use, as well as how impacts on women's time use are associated with changes in women's own and their children's health and nutrition outcomes, using both quantitative and qualitative data. Previous analyses of this programme have demonstrated significant positive impacts on women's empowerment, diets and underweight prevalence (Olney et al., 2016) and on reducing the prevalence of wasting, anaemia and diarrhoea among young children (Olney et al., 2015).

## 2 STUDY SETTING, PROGRAMME DESCRIPTION AND METHODOLOGY

### 2.1 Study setting

The EHFP programme was implemented by HKI in Gourma Province in eastern Burkina Faso. The province is located primarily in the North Sudanian agro‐ecological zone, an arid/semi‐arid environment dominated by rainfed agriculture. Common crops produced in the region include millet, sorghum, maize, groundnuts and cotton. The EHFP programme, and Gourma province generally, is set in the context of a landlocked country that relies on agriculture for about a third of its gross domestic product (GDP). Agriculture employs the majority of the population in the form of subsistence farming, with livestock herding being an additional important source of livelihood and an export product (Etongo, Epule, Djenontin, & Kanninen, 2018). There are important trade and migration routes between Burkina Faso and its neighbouring countries, and many male village residents leave their communities to look for paid work in surrounding countries during the dry season (November–April). The various shifts in governance regimes over the past several decades have led to increased privatization of land and political decentralization, turning control over natural resources (including land) over to local communities. These decentralized governance structures have not been unproblematic, and rural representatives are often accountable to political parties and urban elites rather than local residents, complicating local tenure arrangements (Batterbury, 2010; Gray, 2005). A Rural Land Law was adopted in 2009 and updated in 2012, recognizing customary rights and providing instruments for their formalization, but the complexity of tenure patterns and the institutional changes required prove challenging to the law's implementation (USAID, 2017). It is thus important to recognize the complexity of customary land tenure systems in the country to situate the project discussed in this paper in longer term political and social dynamics.

The programme's intervention area is dominated mostly by the Gourmancema and Zaoga ethnic groups. Similar to groups in other areas in the country (Kevane & Gray, 1999), women's rights to land are indirect, although women can gain access to land through marriage. In certain instances, however, women are granted permission (e.g., by their husbands) to independently farm and control the produce of certain plots. An earlier paper based on the quantitative and qualitative data from the same programme examined in the current paper showed a similar pattern; in this particular area in eastern Burkina Faso, men had control over and ownership of land and other high value agricultural assets. Land was acquired mainly through inheritance (primarily among men) or gifting (primarily among women). Widows could generally only inherit land from their husband under particular circumstances such as having young children or if her husband's family allowed her to. However, data also showed that women controlled their home gardens, small livestock and income generated from these household plots (van den Bold et al., 2015). This is in line with many other customary systems in Africa, where women often have indirect access to land and the produce derived from it through their male relatives, yet often do not have full ownership rights (Kevane & Gray, 1999; Lastarria‐Cornhiel, 1997). Many such access and use rights are determined by customary systems, which rely heavily on family structure, inheritance and marriage practices to determine property rights (Lastarria‐Cornhiel, Behrman, Meinzen‐Dick, & Quisumbing, 2014).

### 2.2 Programme description

In 2010, HKI started the implementation of the two‐year EHFP programme in Gourma Province. The programme was targeted to women with young children (3–12 months of age)^1^ at baseline with the overall goal of improving children's and women's health and nutritional status. The programme was expected to work through three primary pathways: (i) increasing the availability of nutrient‐rich foods through increased household production, (ii) increasing income generation through the sale of surplus production and (iii) improving maternal health and nutrition‐related knowledge and adoption of optimal health and nutrition practices. In addition, it was expected that the programme would increase women's access to and control over resources, such as additional income from the sale of products from home or village production, improved skills, knowledge and self‐confidence in health, nutrition and agriculture gained through the trainings or an increase in bargaining power through the transfer of productive assets. Furthermore, it was expected that changes in gender norms around asset control and ownership could influence the programme's potential impact on agriculture, health and nutrition outcomes. Last, although the programme did not have any specific objectives related to time use, it recognized the potential impacts that women's programme participation could have on how they allocated their time to different activities. Furthermore, it recognized the potential programme impacts on men's time use, through men's involvement in programme related activities such as work in the home garden, or care for children whereas women participated in programme activities.

The programme consisted of two primary components: agricultural production activities and nutrition and health BCC. Agricultural production activities included input distribution (chicks, seeds, saplings and gardening tools) and training in crop and small livestock production. These trainings were carried out in each village by four female village farm leaders (VFLs) at village model farms (VMFs), which functioned as training and demonstration sites for growing vegetables and raising small animals. HKI transferred agricultural inputs and chicks to women at the start of the programme, to encourage them to establish their own homestead food production activities (see also Olney et al., 2015, 2016; Heckert et al., 2019; van den Bold et al., 2015, for detailed information on the programme components).

The BCC component focused on essential nutrition actions (women's nutrition, anaemia prevention and control, iodine intake, prevention of vitamin A deficiency, breastfeeding practices, complementary feeding practices and nutritional care for sick and severely malnourished children) but did not include specific messages on time use (Guyon et al., 2009). Twice a month, women participating in the programme in intervention villages received home visits from community volunteers—either an older women leader (OWL) or a health committee (HC) member—who delivered BCC messages. These two types of volunteers were selected because the effectiveness of the BCC for child health and nutrition outcomes was expected to be potentially different depending on *who* delivered the BCC messages. HC members often delivered nutrition and health interventions and were able to provide links to health services, whereas OWLs were expected to be influential in changing childcare and IYCF practices because of their traditional roles within households and because they are important providers of prenatal and postnatal counselling and child delivery services. Findings from analyses of 2011 process evaluation data showed that HC members were more confident and knowledgeable delivering BCC messages to women than OWLs and more likely to recruit support from other household members (Nielsen et al., 2017). These differences in how HC members and OWLs interacted with households may have had unintended effects on time use patterns.

### 2.3 Study design

**FIGURE 1:**
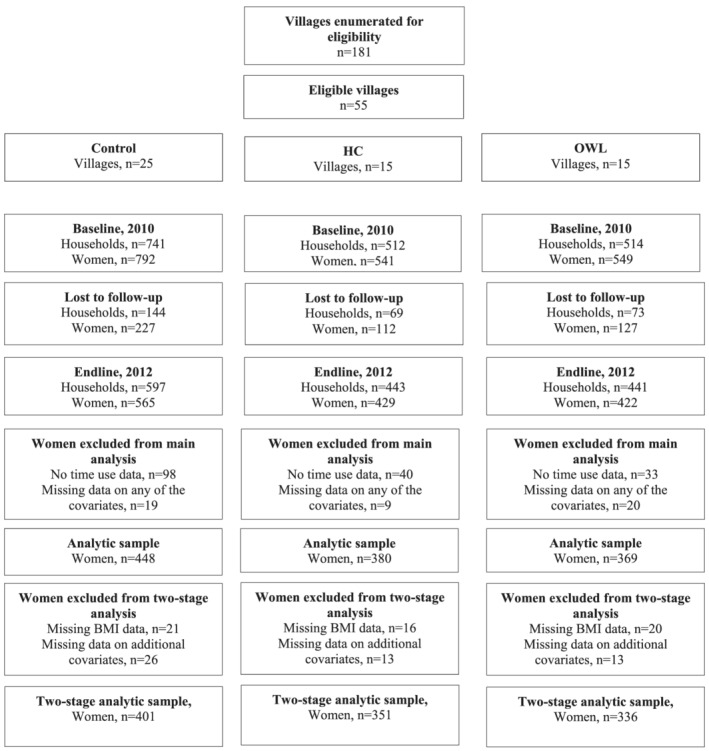
Impact evaluation study flow chart

The EHFP programme was evaluated using a longitudinal cluster‐randomized controlled trial (Olney et al., 2015, 2016), whereby 55 villages that had access to water in the dry season were randomly assigned to one of two intervention groups that both received the agriculture and BCC interventions described above and only differed by who delivered the BCC (OWL [*n* = 15 villages] or HC [*n* = 15 villages]) or a control group (*n* = 25 villages; Figure 1). All households with children 3–12 months of age in 2010 were invited to participate in the programme and/or evaluation. A household was defined as a group of people who usually live together in the same household and share meals. The baseline and endline surveys were carried out between February and May 2010 and February and June 2012, respectively—a period which coincides with the end of the dry season/beginning of the rainy season.

The programme evaluation also included two rounds of qualitative process evaluation conducted in May and June of 2011 and May and June of 2012. For the process evaluation, five households were randomly selected from each of the intervention villages and from 15 of the 25 control villages that participated in the baseline survey. In 2011, two of the five households were selected for more in‐depth semi‐structured interviews (SSIs; Table 1). To the extent possible, the same households that participated in the first round of qualitive research in 2011 were included in the second round of qualitative research in 2012. If a household from the 2011 round was unable to participate in the 2012 round, a replacement household was selected randomly from the list of households that participated in the baseline survey.

**TABLE 1:** Enhanced homestead food production process evaluation study groups and sample sizes (households)

	Intervention villages			Control villages	Total
	OWL	HC	Total		
Process evaluation^a^					
First round (2011)					
Basic semi‐structured interviews	75	75	150	75	225
In‐depth semi‐structured interviews	30	30	60	30	90
Second round (2012)					
Semi‐structured interviews	70	75	145	75	220

^a^ One of the intervention villages dropped out of the EHFP programme before the first round of qualitative research. This meant that the first and second round of the qualitative research (2011, 2012) and the endline survey for the impact evaluation were carried out in a total of 29 intervention villages. The village dropped out due to conflicts within the village, which led to a lack of social cohesion and thereby withdrawal from the programme.

### 2.4 Data collection and measures

As part of the impact evaluation (2010–2012), household surveys were used to collect information on household socio‐economic and demographic characteristics; dwelling characteristics; asset ownership; dietary diversity; participation in health, nutrition, or other social protection programmes; shocks; food security; mother's health and nutrition knowledge; women's status; mother's activities; prenatal and postnatal care; mother's health; feeding practices; and children's health. Clinical assessments of women's and children's nutritional status were also conducted. For anthropometric measures, the weights of women and children were measured using standard assessment methods (Olney et al., 2015, 2016 for more information). Maternal body mass index (BMI) was calculated as weight (in kg) divided by height (in m) squared, and women's underweight was defined as BMI < 18.5 kg/m^2^ (World Health Organization, 2000). For children, height‐for‐age Z‐score (HAZ), weight‐for‐age Z‐score (WAZ) and weight‐for‐height Z‐score (WHZ) were calculated according to the WHO growth reference standards (WHO Multicentre Growth Reference Study Group, 2006). Stunting was defined as HAZ ≤ 2 standard deviations (SDs), wasting as WHZ ≤ 2 SDs and underweight as WAZ ≤ 2 SDs. Child diarrhoea (defined as watery stool in the past week) was measured by maternal recall. Haemoglobin concentration was measured for children from a fingerpick sample using Hemocue. Anaemia was defined as Hb < 11.0 g/dl and severe anaemia as Hb < 7.0 g/dl (World Health Organization, 2011).

In 2010 and 2012, average time spent on agricultural activities (planting, weeding and harvesting) by men and women was based on self‐reported estimates by the manager of each plot. Specifically, the plot manager was asked to estimate the time spent on planting, weeding and harvesting in the past 7 days by men and women in the household. Time use was aggregated into ‘person‐days’, which were calibrated to 6 h of agricultural labour (considered a standard working day). In addition, average time spent on a range of activities (livestock, agriculture, hunting/gathering and child care) and other ‘domestic’ activities (or activities near the home; cooking, laundry, domestic work, purchasing/market activities, collecting firewood and collecting water) by women and men during the 7 days prior to the survey was calculated in hours.

For the two rounds of process evaluation, SSIs were carried out separately with women and their husbands in intervention and control villages, to obtain information on issues related to implementation and utilization of programme components, average time spent per day on the home garden and small livestock production and trade‐offs to participating in the EHFP programme (only asked in intervention villages). In 2012, SSIs were also used to collect information on perceptions on ownership and use of land and agricultural decision making and included an adapted module of the women's empowerment in agriculture index (WEAI) that measured time allocated to productive and reproductive tasks by capturing information on men's and women's activities in 15‐min intervals over the prior 24‐h period (4 am–4 am) (Alkire et al., 2013). The 24‐h module allowed respondents to report on ‘primary’ and ‘secondary’ activities to capture time spent on tasks carried out simultaneously. A primary activity was defined as one on which the respondent was most focused and a secondary activity referred to an activity that was carried out at the same time as the primary one, but that was not the primary objective of the respondent's time use (Alkire et al., 2013). Because the WEAI adapted module was still being developed in 2011, it was only included in the 2012 process evaluation.

### 2.5 Analysis

For this paper, we used a two‐staged analysis to first estimate the impact of the programme on women's and men's time use and second to estimate the association between the change in women's time use with changes in children's and women's health and nutritional status outcomes. The sample sizes for the different analyses varied based on the analysis and included loss to follow‐up and exclusion from the analysis due to missing data specific to each analysis. For details, see Figure 1.

For the first stage—the impact assessment—we used difference‐in‐difference (DID) impact estimates to assess the impact of the EHFP programme on women's and men's time use across different activities. Specifically, we assessed programme impact on the number of person‐days men and women spent on agriculture activities (planting, weeding and harvesting combined) across seasons and the number of hours that men and women spent on domestic tasks in the 7 days prior to the survey. DID impact estimates assessed the change in time use between the baseline (2010) and endline (2012) surveys and controlled for the woman and household head having had any formal education, household size, polygamy, the month when the baseline and endline surveys were administered, and a housing index factor score, constructed using principal components analysis (Olney et al., 2016). Standard errors were clustered at the village level, the unit of randomization. To address potential attrition bias, descriptive statistics and impact estimates for time use were weighted for attrition using inverse probability weights derived from a probit regression predicting the probability to attrit between the baseline and endline surveys. The impact estimates were based on intention‐to‐treat and the *p* values were adjusted to account for testing multiple hypotheses using the Benjamini–Hochberg method (Blakesley et al., 2009).

For the second stage—the association analysis—we assessed the association between the changes in women's time use and changes in maternal and child health and nutrition outcomes for which significant programme impacts were previously found, specifically women's underweight and children's haemoglobin concentration, anaemia, wasting and diarrhoea. For programme impacts on maternal nutrition outcomes, the analysis was conducted using the pooled intervention groups compared to the control group, as the BCC was not expected to differentially affect women's nutritional status outcomes (Olney et al., 2016). However, *who* delivered the BCC was hypothesized to influence child nutritional outcomes, and thus, the analyses for child nutritional outcomes used the individual treatment groups compared to control as was done in the primary analyses (Olney et al., 2015). Programme impacts on child nutrition outcomes were largely limited to the HC group (apart from a significant programme impact on reducing the prevalence of diarrhoea in both the OWL and HC groups). Therefore, the results for the HC group are presented in the main text and those for the OWL group in Appendix A. For the association analysis for changes in women's time use and women's underweight, we controlled for woman and household head education, household size, housing index factor score and woman's age at baseline. An analysis of the associations between women's time use and children's health and nutrition outcomes included these control variables and further controlled for child sex and age at baseline. Associations of time use with health and nutrition outcomes were considered statistically significant at *p* < 0.05 and *p* < 0.01 and were considered to be significantly affected by the programme if both the impact of the programme on a domain of women's time use was statistically significant and the association of the change in time use in that domain was significantly associated with change in a given health or nutrition outcome. Statistical analyses were carried out by LB and GR with guidance from DKO, using STATA version 14 (StataCorp, 2015).

All process evaluation data (2011, 2012) was entered into Microsoft Access and converted to SPSS (Statistical Package for the Social Science) files for analysis (for more details on methodology, see Nielsen et al., 2017; Olney, Behrman, Iruhiriye, Van Den Bold, & Pedehombga, 2013; van den Bold et al., 2015). Data from 2011 were analysed using SPSS version 18; data from 2012 were analysed using SPSS version 19. The data reported on in this paper were coded inductively by MvdB in consultation with DKO, by manually grouping together similar responses and identifying common themes among respondents. Means and standard deviations were calculated for time spent by women and men in intervention and control villages on each category of activity. For the 2012 24‐h time use module, means and standard deviations were calculated for primary and secondary activities combined, primary activities only and secondary activities only. The categories from the original WEAI module were adapted for the purposes of this analysis (Appendix B). Differences in time use patterns as well as time spent on categories of primary and secondary activities were compared between men and women, between women in intervention and control villages and between women who reported time conflicts due to programme participation and women who did not report these time conflicts.

## 3 RESULTS

### 3.1 Programme impacts on time use and associations between changes in women's time use and women's and children's nutritional outcomes

#### 3.1.1 Programme impacts on time use

**FIGURE 2:**
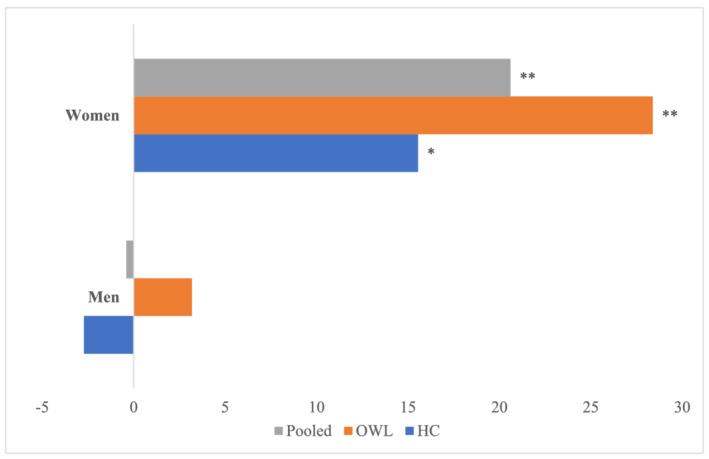
Programme impact on person‐days spent on planting, weeding, harvesting for men and women—full sample. *p < 0.05, **p < 0.01. Comparison is to a control group that did not receive any programme services. All estimates controlled for household size, mother's education, household head's education, housing index factor score, polygamy, baseline and endline survey month and were adjusted for attrition. Standard errors were clustered at the village level

In intervention villages, the average amount of time women spent on household agricultural activities in the 7 days prior to the survey increased by about 4 h over the 2‐year programme period compared to women in control villages (Table 2). In addition, the time women spent on agriculture production (planting, weeding and harvesting) in person‐days across seasons increased by about 21 days between 2010 and 2012 (Figure 2). There were no significant programme impacts on the amount of time women spent on any other activities. Similar results were seen in the HC and OWL villages. Specifically, in HC compared to control villages, the amount of time women spent on agriculture activities increased by about 3 h in the 7 days prior to the survey (Table 3), and the number of person‐days that women spent on planting weeding and harvesting increased by about 16 person‐days (Figure 2). In OWL compared to control villages, women's time spent on agriculture activities and on hunting and gathering in the 7 days prior to the survey significantly increased by about 5 h and about 11 min, respectively. The number of person‐days spent on planting, weeding and harvesting increased by about 28 days over the 2‐year period (Appendix A). There was no programme impact on men's time use (Table 4, Figure 2).

**TABLE 2:** Programme impact on time spent by women in intervention compared to control villages on different activities and association with change in prevalence of women's underweight

Variable	Survey	Full sample	Control	Treatment	DID	Association with women's underweight (%)
*N*		1,242	465	777	1,197	1,088
Time spent on in past 7 days (h)						
Livestock	2010	3.31 ± 5.83	3.65 ± 7.11	3.10 ± 4.84		
	2012	4.52 ± 7.76	3.76 ± 6.43	5.00 ± 8.47	1.52 ± 1.03	0.00 ± 0.00
Agriculture	2010	0.39 ± 2.84	0.25 ± 2.51	0.47 ± 3.03		
	2012	2.61 ± 7.47	0.23 ± 1.43	4.13 ± 9.17	4.03 ± 1.07^+^	0.00 ± 0.00
Hunting/gathering	2010	0.11 ± 1.09	0.19 ± 1.30	0.07 ± 0.93		
	2012	0.02 ± 0.40	0.01 ± 0.19	0.03 ± 0.49	0.12 ± 0.07	0.00 ± 0.00
Childcare	2010	49.83 ± 30.52	50.92 ± 29.29	49.13 ± 31.28		
	2012	29.05 ± 25.07	29.34 ± 24.73	28.87 ± 25.31	−4.58 ± 4.47	0.00 ± 0.00
Total—other domestic activities^a^	2010	36.70 ± 19.09	34.95 ± 17.11	37.81 ± 20.18		
	2012	34.28 ± 24.42	32.21 ± 21.05	35.60 ± 26.27	−0.26 ± 2.25	0.00 ± 0.00

*Note*. All values are coefficient ± SE. Comparison is to a control group that did not receive any program services. All impact estimates controlled for household size, mother's education, household head's education, housing index factor score, polygamy, baseline and endline survey month and were adjusted for attrition. Standard errors were clustered at the village level. To account for multiple comparisons, p‐values for the impact estimates were adjusted using the Benjamini–Hochberg procedure ^+^ *p* < 0.01. Additional controls for association analyses included mother's age at baseline.

^a^ Other domestic activities include time spent on cooking, laundry, domestic work, purchasing/market activities, collecting firewood and collecting water.

**TABLE 3:** Programme impact on time spent by women in health committee villages compared to control villages on different activities and associations with changes in child nutrition and health outcomes

	Survey	Control	HC	DID	Associations with …			
					Haemoglobin	Anaemia (%)	Wasting (%)	Diarrhoea (%)
*N*		465	388		739	739	683	750
Time spent on … in past 7 days (h)								
Livestock	2010	3.65 ± 7.11	3.07 ± 4.99					
	2012	3.76 ± 6.43	4.32 ± 7.06	1.05 ± 1.11	0.01 ± 0.01	0.00 ± 0.00	0.00 ± 0.00	0.00 ± 0.00
Agriculture	2010	0.25 ± 2.51	0.43 ± 2.67					
	2012	0.23 ± 1.43	3.23 ± 8.3	3.39 ± 1.24^+^	0.00 ± 0.01	0.00 ± 0.00	0.00 ± 0.00	0.00 ± 0.00
Hunting/gathering	2010	0.19 ± 1.30	0.10 ± 1.24					
	2012	0.01 ± 0.19	0.02 ± 0.40	0.08 ± 0.09	0.06 ± 0.05	0.01 ± 0.01	0.00 ± 0.01	0.00 ± 0.01
Childcare	2010	50.92 ± 29.29	50.66 ± 31.59					
	2012	29.34 ± 24.73	26.56 ± 22.89	−5.02 ± 5.05	0.00 ± 0.00	0.00 ± 0.00	0.00 ± 0.00	0.00 ± 0.00^**^
Total—other domestic activities^a^	2010	34.95 ± 17.11	38.05 ± 21.40					
	2012	32.21 ± 21.05	33.71 ± 21.69	−0.90 ± 2.68	0.00 ± 0.00	0.00 ± 0.00	0.00 ± 0.00	0.00 ± 0.00

*Note*. All values are coefficient ± SE. Comparison is to a control group that did not receive any programme services. All impact estimates controlled for household size, mother's education, household head's education, housing index factor score, polygamy, baseline and endline survey month and were adjusted for attrition. Standard errors were clustered at the village level. To account for multiple comparisons, *p* values for the impact estimates were adjusted using the Benjamini–Hochberg procedure ^+^ *p* < 0.01. For associations, ^**^ *p* < 0.01. Additional controls for association analyses included child sex, child's age at baseline, mother's age at baseline.

^a^ Other domestic activities include time spent on cooking, laundry, domestic work, purchasing/market activities, collecting firewood and collecting water.

**TABLE 4:** Programme impact on time spent by men in intervention compared to control villages on different activities

Variable	Survey	Full sample	Control	Treatment	DID	OWL	DID	HC	DID
*N*		1,431	583	848		426		422	
Time spent on … in past 7 days (h)									
Livestock	2010	8.67 ± 13.89	8.22 ± 11.42	9 ± 15.45		9.03 ± 14.48		8.97 ± 16.38	
	2012	5.1 ± 9.45	4.69 ± 8.02	5.39 ± 10.36	0.35 ± 1.76	6.26 ± 12.77	1.47 ± 2.35	4.53 ± 7.11	−0.41 ± 1.96
Agriculture	2010	0.62 ± 4.18	0.41 ± 2.95	0.77 ± 4.88		0.98 ± 5.89		0.56 ± 3.59	
	2012	0.84 ± 4.46	0.2 ± 1.35	1.31 ± 5.71	0.93 ± 0.55	1.39 ± 6.14	0.89 ± 0.6	1.23 ± 5.24	0.96 ± 0.63
Hunting/gathering	2010	0.11 ± 0.83	0.15 ± 0.91	0.07 ± 0.76		0.02 ± 0.44		0.13 ± 0.98	
	2012	0 ± 0.08	0.01 ± 0.12	0 ± 0	−0.02 ± 0.05	0 ± 0	0.03 ± 0.04	0 ± 0	−0.06 ± 0.06
Childcare	2010	5.7 ± 13.48	5.36 ± 12.4	5.95 ± 14.21		5.97 ± 14.87		5.93 ± 13.54	
	2012	3.97 ± 8.23	3.59 ± 7.24	4.25 ± 8.89	−0.67 ± 0.9	4.35 ± 9.29	−1.02 ± 1.17	4.14 ± 8.48	−0.44 ± 1.03
Total—other domestic activities^a^	2010	6.77 ± 11.48	6.34 ± 10.36	7.09 ± 12.23		7.08 ± 11.37		7.09 ± 13.05	
	2012	3.02 ± 8.5	3.13 ± 8.92	2.93 ± 8.18	−1.15 ± 0.65	2.61 ± 8.1	−1.56 ± 0.97	3.26 ± 8.26	−0.86 ± 0.76

*Note*. All values are coefficient ± SE. Comparison is to a control group that did not receive any programme services. All estimates controlled for household size, household head's education, housing index factor score, polygamy, baseline and endline survey month and were adjusted for attrition. Standard errors were clustered at the village level. To account for multiple comparisons, *p* values for the impact estimates were adjusted using the Benjamini–Hochberg procedure ^+^ *p* < 0.01.

^a^ Other domestic activities include time spent on cooking, laundry, domestic work, purchasing/market activities, collecting firewood and collecting water.

#### 3.1.2 Associations between changes in women's time use and women's and children's nutritional outcomes

Although the programme significantly increased the amount of time women spent on agriculture activities in both treatment groups, and on hunting and gathering in the OWL group, these changes were not significantly associated with changes in the health and nutrition outcomes for which significant programme impacts were found (Tables 2 and 3). Specifically, the increase in time spent on agriculture was not significantly associated with the change in the prevalence of women's underweight (Table 2). Similarly, for women in both HC intervention villages (Table 3) and OWL intervention villages (Appendix A), an increase in time spent on agriculture activities was not associated with any of the child health and nutrition indicators for which a positive programme impact was found in the primary analyses conducted for this study (Olney et al., 2015), namely, child diarrhoea, wasting, haemoglobin concentration and prevalence of child anaemia. In the OWL intervention group, the increase in time spent on hunting/gathering was associated with a significant decrease in anaemia, but there was not a significant programme impact on anaemia in this group (Appendix A).

### 3.2 Qualitative findings from 2011 and 2012 process evaluation data

#### 3.2.1 Time conflicts due to programme participation

In 2011, women in intervention villages reported that the average amount of time spent taking care of the garden by the main person responsible was, on average, 3.1 h per day (h/d; *SD* 1.8), and 1.6 h/d (*SD* 1.1) on average for other household members (data not shown). Approximately 26% of women (*n* = 15/57) reported that taking care of the garden conflicted with other types of activities such as cooking, watching children and housework. By 2012, the amount of time that the main person and others in the household spent taking care of the garden had declined. Specifically, in 2012, the main person responsible for the garden spent on average about 1.7 h/d (*SD* 0.8) and other household members spent an average of 1.0 h/d (*SD* 0.6) on this. Time related conflicts were also reported by a smaller proportion of women in 2012 with only about 11% (*n* = 13/119) of women in intervention villages in 2012 saying that the time they spent taking care of the garden conflicted with other activities, mainly domestic tasks (e.g., cooking, cleaning the house and child care), outside work (such as going to the market, trading, tending to animals and cutting wood) and agricultural work (such as preparing land for cultivation).

With regard to time spent taking care of poultry, in 2011, women in intervention villages reported that time spent caring for chickens took <1 h/d on average, and ~9% (*n* = 5/58) reported that this activity conflicted with housework. In 2012, there was no change reported in time spent taking care of chickens, and none of the men and women interviewed in intervention villages reported that this interfered with any other activities.

#### 3.2.2 Findings from the 2012 WEAI‐adapted 24‐h time use module

Findings from the 2012 24‐h module revealed several trends. Comparing men's and women's time use showed that women and men had different primary responsibilities, with women in all types of villages spending substantially more time than men on domestic work, as well as on shopping and getting services (such as health services). Although both men and women engaged in paid work and farming, men on average spent significantly more time on these activities, as well as on leisure and social and religious activities (Table 5). Further, defining ‘work’ as the sum of the average amount of hours dedicated to paid *and* unpaid (caring for others, domestic tasks in the home) labour, women in both intervention and control villages worked 2 to 3.3 h more per day than men, even when taking time spent on farming into consideration (Table 5).

**TABLE 5:** Time spent by men and women on primary and secondary activities combined in intervention and control villages

Variable	Women		Men	
	Control	Treatment	Control	Treatment
*N*	73	145	60	116
Time spent on … in past 24 h				
Sleeping and resting	9.1 (3.1)	9.0 (3.0)	8.8 (3.9)	9.0 (2.9)
Eating and personal care	2.0 (1.1)	2.0 (1.1)	2.0 (1.1)	2.1 (1.2)
Working	6.5 (2.8)	5.7 (4.2)	7.0 (4.1)	6.7 (4.9)
Farming	5.5 (2.4)	4.4 (2.2)	6.6 (2.9)	6.0 (2.6)
Shopping and purchasing services (including health services, laundry, haircut, etc.)	4.4 (2.1)	5.1 (3.0)	2.6 (1.8)	3.7 (1.7)
Textile‐related work	1.8 (0)	2.1 (1.1)	3.0 (0)	3.9 (4.7)
Domestic tasks	5.7 (2.4)	5.1 (2.6)	3.0 (2.3)^a^	2.3 (1.9)
Care for others	1.1 (0.6)	1.0 (0.7)	^b^	0.8 (0.4)
Travelling and commuting	3.4 (1.8)	3.3 (3.2)	4.2 (3.6)	3.3 (2.7)
Leisure, social and religious activities	3.5 (2.9)	3.9 (3.4)	5.2 (4.0)	6.4 (4.2)

*Note*. Numbers are mean (*SD*).

^a^ Does not include average time spent on cooking (data missing).

^b^ Data on time spent on care by men in control villages was unavailable due to lack of response to this particular question.

Comparing time use patterns between women in intervention and control villages revealed only slight differences in time use patterns (0.1–1.1 h/d), with women in intervention villages spending *less* time on average than those in control villages on paid work, farming and domestic tasks, but more time on shopping/purchasing services, textile‐related work; and leisure, social and religious activities, while spending a similar amount of time on care for others. Women in both types of villages spent most of their time working between 5.7 h/d (intervention villages) and 6.5 h/d (control villages). This was followed by farming for women in control villages (5.5 h/d) and shopping/purchasing services for women in intervention villages (5.1 h/d; Table 5). In general, women in intervention villages reported *more* secondary activities than any other group, and these included (paid) work; textile‐related work; and leisure, social and religious activities. Care for others was hardly reported as either a primary or secondary activity by women or men in either type of village.

Finally, data from the 24 h time use module were compared between women in intervention villages who reported time use conflicts in 2012 (11%; *n* = 13/119) and those who did not. Those who reported that the EHFP programme participation affected other activities on average spent slightly *more* time on sleeping and resting; eating and personal care; domestic tasks (except cooking); and own business work compared to those who said that programme participation did *not* conflict with other activities. Women in intervention villages who reported *no* time use conflicts on average spent slightly *more* time on farming and at least 6 h *more* on leisure, social and religious activities than those who reported time use conflicts (Appendix C). Interestingly, women in intervention villages who reported time use conflicts hardly reported any secondary activities (data not shown).

## 4 DISCUSSION

The above analysis shows that the EHFP programme led to a significant increase in time spent on agricultural activities among women in intervention compared to control villages. In the OWL group, the programme also had a significant programme impact on increasing the time spent on hunting and gathering. There were no significant programme impacts on time spent on other activities by women or on time spent on any activities by men. In OWL villages, an increase in time spent on hunting/gathering was associated with a decrease in anaemia prevalence; however, in this treatment group, there was no significant impact on child anaemia (Olney et al., 2015). Thus, overall, the two‐stage analyses showed that the increase in the time women spent on agriculture activities was not associated with changes in women's or children's nutritional outcomes on which there were programme impacts in the HC or OWL groups (Olney et al., 2015, 2016).

The results from the 2011 and 2012 process evaluation support the findings from the two‐stage analyses. The process evaluation results also support findings in earlier work on time use (e.g., Johnston et al., 2018), showing that women generally work longer hours than men per day, taking into account both productive and reproductive activities, and that women generally spend more time on domestic work than men, whereas the latter spend more time on paid work and farming. When comparing time use patterns, women in intervention villages reported spending less time than women in control villages on domestic responsibilities, possibly due to involvement in the EHFP programme. In addition, women in intervention villages reportedly spent less time than women in control villages on (paid) work and farming in the past 24 h. This suggests that, on a given day, their participation in the EHFP programme did not substantially increase their time allocated to agricultural activities. It could also suggest that they adjusted their expectations about workload over time or did not perceive their time use on agriculture to have increased by much. It is also possible that they have become accustomed to the agricultural activities and have become more efficient in undertaking them.

The comparison of time use patterns between women in intervention villages who reported time use conflicts and those who did not, albeit based on a small sample, does not indicate compromises on personal care, domestic tasks, and caring for others, even though women in intervention villages who reported time use conflicts reportedly spent less time on farming than women in those same villages who did not report time use conflicts. The increased age of children over time may also have meant that women experienced fewer time conflicts and were able to engage in more activities with an older child. Furthermore, although there was also a reported reduction in time spent taking care of poultry between 2011 and 2012 by women in intervention villages, this could also be due to women having become accustomed to the poultry over a period of 1 to 2 years and hence not having to make substantial time adjustments to care for poultry by 2012. Data from the 2012 24‐h time use module does not point to strong conclusions regarding women's time poverty due to programme participation, results that are also supported by the findings from the quantitative analysis.

Taken together, the results from the quantitative impact evaluation and the two rounds of qualitative process evaluation data show that increases in time spent on agricultural activities by women in the programme were not associated with changes in their own or their children's health and nutritional outcomes. Thus, the increased time spent on agriculture activities does not seem to outweigh the overall positive programme impacts on indicators such as agricultural production, women's health and nutrition related knowledge, women's ownership of—and decision‐making power over—agricultural assets and small animals, perceptions regarding women's land rights (van den Bold et al., 2015), women's underweight and empowerment measures (Olney et al., 2016; van den Bold et al., 2015), and prevalence of child anaemia, wasting and diarrhoea (Olney et al., 2015). Furthermore, positive impacts on the above intermediary outcomes such as women's knowledge and empowerment may have mediated any potential negative impacts of the increase in women's time spent on agricultural activities on women's and children's health and nutrition outcomes.

There are four main caveats that should be taken into consideration when interpreting these data. First, we focused primarily on women's and men's time use. Although the qualitative data include time spent by ‘other household members’ on taking care of the garden and chickens, we did not have data on how much time other household members spent on taking care of others, cooking or other domestic tasks, nor which activities are shifting to other household members. The total time spent on these activities may therefore in reality be higher. Second, time use patterns are influenced by seasonality. Because time use data were collected during the transition from the harvest to the ‘hungry’ season in Gourma Province, it is unclear how time allocation at this time of year is different from time allocated in another time of year, and the data may in fact reflect relatively less time spent on agricultural activities. Third, we did not measure work intensity, which has also been highlighted as an important shaper of productive and reproductive labour (Stevano et al., 2018). This could mediate some of study findings, for example, where women who reported time use conflicts may have had a more intense work schedule, focused on their primary activities rather than undertaking more secondary activities, and took more time for rest. Fourth, because the data were self‐reported, it is possible that actual time use may differ from what was reported, or that reporting on time use spent on agricultural or home garden activities is inflated due to the expectation of programme participation.

In conclusion, how women allocate their time is a potentially important factor mediating the impact of agricultural interventions on maternal and child health and nutrition outcomes (Johnston et al., 2018; Stevano et al., 2018). In this particular context in western Burkina Faso, this was not the case. Although the EHFP programme increased the amount of time that women spent on agriculture, this did not appear to have negative effects on maternal or child health and nutrition outcomes. Future research on the perceived and actual impacts of nutrition‐sensitive agriculture programmes on health and nutrition outcomes of women, children and other household members and the mediating effect of changes in time use patterns is needed and would contribute substantially to the relatively limited evidence base. Given the importance of seasonality in agricultural production, time use data should be collected more than once a year, to better understand how seasonality affects time use (Blackden & Wodon, 2006; Stevano et al., 2018) and associations with maternal and child health and nutrition outcomes. Women's time use also needs to be placed in the context of other household members' time use patterns (Stevano et al., 2018).

Going beyond the context of this specific intervention, it is furthermore important to place these studies in their historical and political contexts, in order to situate gender and food security challenges in relation to other factors such as education opportunities, stability of livelihoods options (including both formal and informal employment opportunities for both women and men) and measures of social differentiation such as class. It is also critical to ensure that these kinds of analyses do not result in the reproduction of ideas about gendered divisions of labour that put reproductive (and unpaid) responsibilities squarely on the shoulders of women. Future nutrition‐sensitive agriculture programmes should thus pay careful attention to how intervention design and targeting can avoid such unintended effects.

## CONFLICTS OF INTEREST

No potential conflict of interest was reported by the authors. All authors have approved the final version of this paper.

## CONTRIBUTIONS

MvdB was the lead author of this paper and led the qualitative analyses and drafting of the paper. MvdB, DKO, AQ and LB were responsible for the conceptualization of the paper, providing inputs into the analyses and writing, and for reviewing the manuscript. LB and GR conducted the quantitative analysis under the guidance of DKO. DKO was one of the co‐PIs for the evaluation of the EHFP programme in Burkina Faso (along with Andrew Dillon) and hence played an important role in initiating and guiding this paper. AQ played an advisory role as a coprincipal investigator of GAAP with extensive expertise on gender, agriculture and nutrition research; she also contributed to the finalization of the paper. AP and MO were responsible for collecting the data used for this paper as part of HKI and served in an overall advisory role on the paper.

## ENDNOTE

1
For consistency, we refer to ‘women’ throughout the paper, but it is important to note that all women participating in the programme were mothers. Similarly, we refer to ‘men’, recognizing that all men in the programme were fathers.

## APPENDIX A

Programme impact on time spent by women on different activities in older women leader compared to control villages and associations with changes in prevalence of child nutrition outcomes

**Table:**

Variable	Survey	Control	OWL	DID	Associations with …			
					Haemoglobin	Anaemia (%)	Wasting (%)	Diarrhoea (%)
*N*		466	389		757	757	704	760
Time spent on … in past 7 days (h)								
Livestock	2010	3.65 ± 7.11	3.13 ± 4.69					
	2012	3.76 ± 6.43	5.68 ± 9.64	2.25 ± 1.42	0 ± 0.01	0 ± 0	0 ± 0**	0 ± 0
Agriculture	2010	0.25 ± 2.51	0.5 ± 3.36					
	2012	0.23 ± 1.43	5.03 ± 9.89	5 ± 1.38^+^	0 ± 0.01	0 ± 0	0 ± 0	0 ± 0
Hunting/gathering	2010	0.19 ± 1.3	0.04 ± 0.43					
	2012	0.01 ± 0.19	0.03 ± 0.57	0.18 ± 0.07^+^	0.1 ± 0.05^*^	−0.02 ± 0.01^*^	0 ± 0.03	0.01 ± 0.02
Child care	2010	50.92 ± 29.29	47.6 ± 30.93					
	2012	29.34 ± 24.73	31.17 ± 27.35	−3.91 ± 5.76	0 ± 0	0 ± 0	0 ± 0^**^	0 ± 0^*^
Total—other domestic activities^a^	2010	34.95 ± 17.11	37.57 ± 18.89					
	2012	32.21 ± 21.05	37.48 ± 30.08	0.72 ± 2.71	0 ± 0	0 ± 0	0 ± 0	0 ± 0

^a^ *Note*. All values are coefficient (SE). Comparison is to a control group that did not receive any programme services. All impact estimates controlled for household size, mother's education, household head's education, housing index factor score, polygamy, baseline and endline survey month and were adjusted for attrition. Standard errors were clustered at the village level. To account for multiple comparisons, *p* values for the impact estimates were adjusted using the Benjamini–Hochberg procedure ^+^ *p* < 0.01. For associations, ^*^ *p* < 0.05, ^**^ *p* < 0.01. Additional control variables for the association analyses included child sex, child's age at baseline, mother's age at baseline.

^a^ Other domestic activities include time spent on cooking, laundry, domestic work, purchasing/market activities, collecting firewood and collecting water.

## APPENDIX B

Categories of activities used in 2012 adapted women's empowerment in agriculture index (WEAI) time use module^a^

**Table:**

Adapted categories		Original categories used in the WEAI^b^	
A	Sleeping and resting	A	Sleeping and resting
BC	Eating and personal care	B	Eating and drinking
		C	Personal care
D	School	D	School (also homework; no data)
EF	(Paid) work	E	Work as employed (excludes commuting to and from work)
		F	Own business work (except farming, fishing and textile work)
G	Farming	G	Farming/livestock/fishing (primarily small‐scale for own consumption and for selling)
J	Shopping/getting services (including health services)	J	Shopping/getting services (including health services)
K	Textile‐related work	K	Weaving, sewing, textile care (for selling and own consumption)
LM	Domestic tasks	L	Cooking
		M	Domestic work (including fetching wood and water)
N	Care for others	N	Care for children/adults/elderly (unpaid)
P	Travelling and commuting	P	Travelling and commuting
QTUW	Leisure, social, and religious activities	Q	Watching TV/listening to radio/reading (excludes for homework or work)
		T	Exercising
		U	Social activities and hobbies
		W	Religious activities
X	Other activities	X	Other, specify

^a^ Categories used in the original WEAI were combined and adapted in the 2012 process evaluation module. This was done in consultation with HKI staff and co‐authors.

^b^ Source: Alkire et al., 2013.

## APPENDIX C

Time use (2012) reported by women in intervention villages who did and did not experience time conflicts due to programme participation

**Table:**

Activity	Women who reported time conflicts due to programme participation	
	Yes (*N* = 13)	No (*N* = 106)
In past 24 h, h spent on		
Sleeping and resting	9.5 (3.1)	8.8 (3.0)
Eating and personal care		
Eating	1.4 (0.8)	1.3 (0.8)
Personal care	0.8 (0.5)	0.7 (0.4)
School		
Working		
As employed		7.1 (5.8)
Own business	8.5 (2.8)	5.4 (4.5)
Farming	3.8 (2.0)	4.4 (2.2)
Shopping/getting service		6.4 (1.7)
Textile‐related work		1.6 (0.7)
Domestic tasks		
Cooking only	2.0 (0.9)	2.4 (1.1)
Domestic work	3.8 (2.0)	3.2 (2.2)
Care for others	1.0 (0.3)	1.0 (0.7)
Travelling and commuting	1.1 (0.4)	3.8 (3.6)
Leisure, social and religious activities		
Watching TV/listening to radio/reading		2.1 (1.9)
Exercising		2.3 (0.7)
Social activities and hobbies	2.3 (1.2)	3.9 (3.5)
Religious activities	0.9 (0.4)	1.2 (1.0)

*Note*. Numbers reported are mean (*SD*). Table shows primary and secondary activities combined.
